# Perceptions and Practices towards Medication Non-Adherence among Hypertensive Patients: An Observational Study

**DOI:** 10.7759/cureus.5917

**Published:** 2019-10-15

**Authors:** Muhammad N Khan, Najia Soomro, Tariq Ashraf, Khalid Naseeb, Rajesh Kumar, Usman Bhatti, Syed Taha Ali, Musa Karim

**Affiliations:** 1 Interventional Cardiology, National Institute of Cardiovascular Diseases (NICVD), Karachi, PAK; 2 Cardiology, Liaquat National Hospital, Karachi, PAK; 3 Cardiology, National Institute of Cardiovascular Diseases (NICVD), Karachi, PAK; 4 Department of Biostatistics, National Institute of Cardiovascular Diseases (NICVD), Karachi, PAK

**Keywords:** non-adherence, hypertension, blood pressure, perceptions, medication

## Abstract

Background

The present study broadly evaluates the adherence to pharmacotherapy, perceptions, and practices among ambulatory hypertensive patients attending a cardiac institution in Karachi, Pakistan.

Methods

A cross-sectional, single-center study was conducted at the National Institute of Cardiovascular Diseases Karachi, Pakistan. The study continued from 4 July 2019 to 3 September 2019. A total of 200 patients with a primary diagnosis of hypertension (HTN) were recruited for the study. The data was collected through a questionnaire based on a nine-item modified adherence predictor scale to assess medication adherence. Along with the demographic details patients smoking status, history of comorbidities and past complications were noted.

Results

It was found that the mean age of the study population was 56.45±12.36 years. A total of 62.5% of patients were taking medication daily while 15.5% were consuming medications intermittently and only 6.5% patients were not adherent at all. Around 35% patients preferred follow-up visits once in a month. Besides this, 35.5% patients never monitored their blood pressure while more than half of the studied population believed that their BP has mostly been controlled and skipped the prescribed medication.

Conclusion

The study indicated that the perception and awareness among the hypertensive patients regarding their medical condition are suboptimal. Concerted strategies like health education program and campaigns must be launched in order to help the sufferers.

## Introduction

Hypertension (HTN) is one of the major attributable risk for the cardiovascular morbidity and mortality and hence it makes up more than half of the global disease burden causing numerous premature deaths per annum [1-2]. Research studies indicate that blood pressure (BP) management helps to reduce the disease intensity and risk of other comorbidities like stroke, renal disease, heart failure, and death [3-4]. Moreover, the disease risk and associated death ratio are more commonly observed in low to middle-income countries, with increased prevalence among individuals having lower socioeconomic statuses [5-6]. According to the estimates provided by the World Health Organization (WHO), each year about 17.3 million lives are carried away by cardiovascular diseases and by 2030 it is predicted that the rate might increase to 23 million [7]. Also, one-quarter of the adult population is affected by this chronic non-communicable disease worldwide. Pakistan being on top of the list contributing 50% of the affectees to this global burden, being a developing state with compromised healthcare facilities the diagnosis and treatment of such health risks are infrequent [7]. 

Among numerous influencers those are associated with HTN, advancing age is one of the most significant non-modifiable risk factor [8]. However, gender is another lead with greater susceptibility in men at an early age while in women, developing HTN is more after the age of 60 years [9]. Followed by race as the other influencer to increase the risk of HTN with reported higher prevalence among the black population when compared to white with mechanism still unknown. Other than that, there can be an influence of family disposition, decreased physical activity, increased weight, smoking, alcohol consumption as well as the precipitating influence of dietary factors such as high sodium, low potassium, and decreased Vitamin D consumption. Among the serious complexities’ cholesterol, diabetes, sleep apnea, and kidney diseases might play the role of triggering factors.

Even though the field of medication has advanced and flourished rapidly, still HTN is poorly controlled among the diagnosed patients [10]. It may be due to poor medication adherence, non-compliance with follow-up visits, and lifestyle modifications [10]. Medication non-adherence is the prime difficulty that must be identified and addressed in each case. Around 50% of the patients suffering from chronic illnesses are adherent to antihypertensive medications prescribed by the healthcare provider [11]. It is recommended that moderating the salt and fat intake, regular exercising, reducing stress as well as quitting the habits of alcohol consumption and smoking can keep BP quite under optimal range [12-13]. Furthermore, it also reduces the risk of having a stroke, myocardial infarction, or heart failure [14]. For treatment adherence and long-term effectiveness, self-management is necessary along with the self-information of the patient about their clinical condition that should be well understood by the patient and assessed regularly [15]. Though due to long-term morbidity, the patient can develop coping skills to combat the disease more appropriately with active involvement in disease handling, which is a progressive approach [16].

Therefore, lifestyle modification and treatment adherence are the two prime factors that must be keenly planned for the effective management of HTN [10]. The primary aim of the study was to evaluate medication adherence and the contributing factors of HTN along with the assessment of common perceptions and practices related to its management.

## Materials and methods

This cross-sectional, single center study was conducted at the cardiology clinic of tertiary care hospital, National Institute of Cardiovascular Diseases Karachi, Pakistan. The study continued for a period of three months from 4 July 2019 to 3 September 2019. A total of 200 patients with a primary diagnosis of HTN were recruited for the study. Patients on antihypertensive medications for at least three months were enrolled irrespective of the gender using consecutive sampling technique. While patients under hypertensive emergency with BP > 180/110 mmHg or those with acute coronary syndrome, acute stroke, hypertensive encephalopathy, aortic dissection, or acute kidney injury, uncontrolled psychiatric disorder (schizophrenia or major depression), stage V chronic kidney disease (CKD) or end-stage renal disease (glomerular filtration rate < 15 ml/min/1.73 m^2^) were excluded. Moreover, patients with a history of active substance abuse, patients who refused to take medications, and pregnant female patients were excluded from the study sample.

The data were collected through a questionnaire based on components of the nine-item modified Morisky Adherence Predictor Scale (MMAPS), to assess medication adherence, patients’ practices, and perception about HTN and BP outcomes [17]. HTN status was marked as controlled and uncontrolled BP. Systolic blood pressure (SBP)>140 or diastolic blood pressure (DBP)> 90 was considered as uncontrolled BP, else it was considered as controlled. The patient demographic details were collected along with smoking status, history of comorbidities such as obesity, diabetes, and high cholesterol and history of past complications like stroke, kidney failure, visual problem, and heart attack or failure.

Before administering the study questionnaire, informed consent was obtained from each patient (both in Urdu and English). All conducts were performed ethically in accordance with the International Conference on Harmonization - Good Clinical Practice (ICH-GCP) guidelines and the ethical approval was received from the Ethical Review Committee of National Institute of Cardiovascular Diseases (Ref. No. ERC-39/2019) prior to the study.

The collected data were analyzed using International Business Machines Statistical Package for the Social Sciences (IBM SPSS), Version 21.0, qualitative data were summarized as frequency and percentage while mean ± standard deviation was used for quantitative data. The association of gender, smoking status, education, comorbidities, and past complications with the HTN status was evaluated through the Chi-square test. Mann-Whitney U-test was used to assess the relationship between patient’s practices, perception, and HTN status where p value <0.05 was considered significant.

## Results

A total of 200 patients fulfilling the eligibility criteria and provided consent. Out of which 124 were males and 76 were females. The mean age of the study participants was 56.45±12.36 years with a mean duration of HTN 5.80 ± 6.43 years (Table 1). The clinical and personal history was also recorded, i.e., 85 out of the total were smokers, 143 had a sedentary lifestyle, and 76 were overweight. The information regarding comorbidities was also taken and it was found that 122 patients were diabetic, 154 with high cholesterol, 140 reported visual problems, and 169 had the previous history of heart attack/failure. Based on the criteria mentioned in the above methodology, 73 out of 200 patients were observed with controlled BP while 127 had uncontrolled BP (Table 1).

**Table 1:** Demographics and clinical characteristics of the study population *BP: Blood Pressure; HTN: Hypertension; SBP: Systolic Blood Pressure; DBP: Diastolic Blood; BMI: Body Mass Index *Quantitative data were presented with mean ± standard deviation; Qualitative data were presented with frequency and percentage.

Variables	Sub-categories	(n=200)
Age (Years)		56.45±12.36
Duration of HTN (Years)		5.80±6.43
SBP (mmHg)		150±27.99
DBP (mmHg)		85.70±12.74
Gender	Male	124(62.0)
	Female	76(38.0)
Education	No Education	87(43.5)
	Primary	38(19.0)
	Secondary (Metric)	40(20.0)
	Intermediate	13(6.5)
	Bachelors	17(8.5)
	Masters	5(2.5)
Smoker	Yes	85(42.5)
	No	115(57.5)
HTN Status	Controlled BP	73(36.5)
	Uncontrolled BP	127(63.5)
Lifestyle	Active	57(28.5)
	Sedentary	143(71.5)
BMI Index	Underweight	5(2.5)
	Normal	119(59.5)
	Over weight	76(38)
Other chronic illnesses	Diabetes	122(61.0)
	High Cholesterol	154(77.0)
	Stroke	17 (8.5)
	Kidney failure	26 (13.0)
	Visual problem	140 (70.0)
	Heart attack/failure	169 (84.5)

According to the results in Table 2, 62.90% (78/124) of males and 64.47% (49/76) of females were having uncontrolled BP, out of 127 patients from the uncontrolled BP group 40.15% were smokers while 46.67% of the 73 patients in controlled BP group were smokers. A significant association was observed between obesity and HTN status (p<0.05), and 65.79% (50/76) overweight patients were having controlled BP. Similarly, diabetes and visual problems also had a significant association with HTN status. Moreover, increased comorbidities were observed among the patients having uncontrolled BP.

**Table 2:** Association of hypertension status with sociodemographic characteristics of the study population *BP: Blood pressure; BMI: Body Mass Index *SBP > 130 or DBP > 80 was considered as uncontrolled BP; *Chi-square was applied; p value<0.05 was considered significant.

Variables	Sub-category	HTN status		P-value
		Controlled (n=73)	Uncontrolled (n=127)	
Gender	Male	46(63.01)	78(61.4)	0.823
	Female	27(36.9)	49(38.58)	
Smoking Status	Smoker	34(46.57)	51(40.15)	0.377
	Non-smoker	39(53.42)	76(59.84)	
BMI Index	Underweight	5(6.84)	0(0)	0.023
	Normal	42(57.53)	77(60.62)	
	Over weight	26(35.61)	50(39.37)	
Other Chronic Illnesses	Diabetes	37(50.68)	85(66.92)	0.023
	High cholesterol	58(79.45)	96(75.59)	0.532
	Stroke	6(8.21)	10(7.87)	0.894
	Kidney failure	10(13.69)	16(12.59)	0.824
	Visual problem	41(56.16)	99(77.95)	0.001
	Heart attack/failure	60(82.19)	109(85.82)	0.874
Life style	Sedentary	51(69.86)	92(72.44)	0.697
	Active	22(30.13)	35(27.55)	

Participants' perceptions and practices regarding their hypertensive illness showed that 125/200 were taking medication daily while 13/200 were those who never took prescribed medication. Follow-up frequency was also monitored, 70/200 preferred follow-up visit once per month while 71/200 never went for a follow-up visit. BP monitoring frequency was also recorded 20/200 monitored BP daily while 71/200 never monitored their BP. 100/200 believed that BP is mostly controlled and 40 disagreed to the point moreover, 64/200 agreed that BP medication is necessary while 25/200 disagreed (Table 3).

**Table 3:** Perceptions and practices of hypertensive patients regarding blood pressure management *BP: Blood pressure

Variables	Sub-categories	n(%)
Medication Intake	Everyday	125 (62.5)
	Most of the days	31 (15.5)
	Some of the days	31 (15.5)
	Never	13 (6.5)
Follow-up Visits	Multiple times in a month	11 (5.5)
	Once in a month	70 (35.0)
	Once every 6 months	21 (10.5)
	Once a year	27 (13.5)
	Never	71 (35.5)
BP Monitoring Frequency	Everyday	20 (10.0)
	Every 2-3 days	40 (20.0)
	Once a week	22 (11.0)
	Once a month	47 (23.5)
	Never	71 (35.5)
BP is mostly controlled	Strongly agree	4 (2.0)
	Agree	100 (50.0)
	Uncertain	41 (20.5)
	Disagree	40 (20.0)
	Strongly disagree	15 (7.5)
BP medication is necessary	Strongly agree	55 (27.5)
	Agree	64 (32.0)
	Uncertain	43 (21.5)
	Disagree	25 (12.5)
	Strongly disagree	13 (6.5)

There was a significant association observed between medication intake frequency and HTN status (p<0.05). Moreover, it was also significantly associated with perceptions of hypertensive patients related to the illness. Perceptions like BP is mostly controlled and BP medication necessity was also positively associated (p<0.05) (Table 4).

**Table 4:** Patients’ practices and perception in relation to hypertension status *HTN: Hypertension; BP: Blood pressure *Mann–Whitney U-test was applied keeping P-value <0.05 as statistically significant cutoff

Variables	HTN Status Mean Rank		P-value
	Controlled	Uncontrolled	
Medication Intake Frequency	137.19	79.41	0.000
Follow up visit Frequency	99.58	101.03	0.857
BP monitoring Frequency	109.84	95.13	0.073
BP is mostly controlled	113.18	93.21	0.011
BP medication is necessary	137.84	79.04	0.000

No association was observed between medication adherence and the educational level of the patient. The association of medication adherence status with educational level of the patient is presented in Table 5.

**Table 5:** Association of medication adherence status with the educational level of patient Non-adherent = “some of the days” or “never” Adherent = “every day” or “most of the days” *Chi-square was applied; P-value<0.05 was considered significant

Educational Level	Base [N]	Medication Adherence Rate		P-value
		Non-adherent	Adherent	
Illiterate	87	18(20.69)	69(79.31)	0.958
Primary	38	8(21.05)	30(78.95)	
Secondary to intermediate	53	13(24.53)	40(75.47)	
Bachelors or higher	22	5(22.73)	17(77.27)	

## Discussion

The current study provides several important insights into the perceptions and practices of the patients towards HTN management. The major recognizable features associated with uncontrolled HTN were gender, smoking, marital status, diabetes, medication adherence, etc. It is a known fact that lack of treatment adherence leads to increased hypertensive severity and boosts the mortality rate [18]. An optimal medication intake frequency was observed in the study sample, 62.5% of patients were taking medication daily while 15.5% showed intermittent adherence and 6.5% never took the prescribed medication (Table 3). It is evident that following the prescribed treatment regime and attending the follow-up visits results in improved health outcomes and decreases the overall healthcare costs [19-20]. According to the previous literature more than half of the hypertensive patients discontinue medication roughly a year after initiation [21]. The most highlighted factor found in association with the treatment non-adherence are the side-effects of the medication [12].

Though it has been revealed that low literacy rate is one of the leading aspects behind poor treatment adherence [22]. But according to our findings, most of the HTN patients enrolled in the study were adherent to medication (Table 3), which in comparison to their educational status was contradictory as many of them had below-average literacy rate (Table 5). Around 43.5% were illiterate while only 20% had passed the secondary education (Table 1). Several unintentional reasons such as forgetfulness, decreased health literacy, lack of symptoms and carelessness are also associated with medication non-adherence [23]. Hence, it is the prime responsibility of the healthcare providers to prevent non-adherence either through reflective listening or by considering the patient’s psychosocial and medical requirements keeping personal opinions aside.

A significant association was observed between medication adherence and HTN status (Table 4), as other parallel studies also specified a direct relationship between treatment adherence and outcomes of HTN [24]. Research indicates that the patient’s perception and behaviors are dependent upon their belief regarding disease susceptibility, disease severity and the benefits they are willing to attain [25]. Our results showed that more than half of the population agreed that BP medication is necessary (Table 2). Likewise, another study reported that 90% patients believed antihypertensive medications were necessary to keep their BP under normal range, while there were a few who believed that it is a benign illness and no therapeutic help is required for recovery [19]. Despite being diagnosed with high BP, more than 70% of the Asian hypertensive patients have uncontrolled BP [26]. Reluctance and negative perceptions have taken over the disease management rate. Around 50% of the study patients believed that their BP is mostly controlled and this negative perception holds a significant impact on HTN status (p<0.05) (Tables 3 and 4).

It was observed that 5.5% patients visited the healthcare provider for follow-up multiple times in a year, 13.5% preferred follow-up visit once in a year while 35.5% patients never went for the follow-up visits (Table 3). Follow-up visits have the utmost importance in the life of hypertensive patients, the risk of multimorbidity and mortality increases due to the untracked visit-to-visit variability (VVV) of BP [27]. Secondary to the follow-up frequency, BP monitoring frequency was also observed, 35.5% of patients never monitored their BP while only 10% were consistently monitoring their BP (Table 3). Moreover, no significant association was observed between the monitoring frequency and HTN status (p=0.073) (Table 4). Similarly, Ramón and his colleagues concluded in their study that the mean BP values are linked with the duration of measurement as compared to the frequency of measurement [28].

According to the National Institute of Diabetes and Digestive and Kidney Diseases Health Information Center, increased weight leads to numerous health problems mainly associated with heart [29]. BMI holds significant association with the HTN status either controlled or uncontrolled, 39.37% overweight patients had uncontrolled BP (Table 2). Centers for Disease Control and Prevention (CDC) report states regarding obesity and BP states that increased weight intensifies the risk of high BP disposing an individual to several other co-morbidities.

Our study explored medication adherence, perceptions, and practices among hypertensive patients. There are few limitations associated with it, first and the most significant is self-reporting of medication adherence, although MMAPS was used to assess compliance, other is the high prevalence of diabetes and coronary artery disease which may be overestimated as most of these patients have multiple comorbid conditions. Moreover, it was a uni-center study, the sample was limited to a single tertiary healthcare center only. There could be a more pronounced display of patient’s perception and practices regarding the illness. Therefore, further research is required to explore other associated factors with HTN to fill gaps in the existing literature.

## Conclusions

This study explored the association of negative perception regarding controlled BP and HTN status. Majority of the patients believed that antihypertensive medication is necessary for effective BP control. It is suggested that treatment adherence education and counseling for hypertensive patients are necessary in order to ensure improved BP control and therapeutic outcomes.
